# Creating web applications for online psychological experiments: A hands-on technical guide including a template

**DOI:** 10.3758/s13428-023-02302-2

**Published:** 2023-12-05

**Authors:** Gáspár Lukács, Erwin Haasnoot

**Affiliations:** 1 Department of Cognition, Emotion, and Methods in Psychology, Faculty of Psychology, University of Vienna, Liebiggasse 5, A-1010 Vienna, Austria; 2 Data Science Group, Faculty of Electrical Engineering, Mathematics and Computer Science, University of Twente, Enschede, Netherlands

**Keywords:** Online experiment, Research method, Guide, Web application, JavaScript

## Abstract

The present tutorial provides a technical overview of how to create web applications for online psychological experiments from scratch via the HTML/CSS/JavaScript framework. This approach allows virtually unlimited flexibility in accomplishing anything in an online experiment that a regular computer (or smartphone, etc.) is capable of. Apart from offering a fast introduction for complete beginners, this tutorial may also serve as a helpful guideline for more experienced programmers and researchers. Connected to the tutorial, a specific implementation is also given via the free and open-source template project at https://github.com/gasparl/expapp, intended to be improved by the community to always follow the latest technological advancements and general good practices.

The dramatic increase and impact of behavioral research conducted via the Internet in the past decades, and in particular in the past years (e.g., Anwyl-Irvine et al., 2021; Peer et al., 2021), also already led to several notable papers providing general abstract overviews of how to create online experiments (e.g., Gagné & Franzen, 2023; Grootswagers, 2020; Kochari, 2019; Woods et al., 2015). However, none of these provide the details of how exactly an online experiment should be implemented at the technical level. The present paper is intended to fill in this gap as a hands-on tutorial for implementing web applications (web apps) for online psychological experiments (from here on: ExpApps) in the HTML/CSS/JavaScript (JS) web stack.^1^

Many ExpApp creator frameworks (e.g., *PsychoJS*, Peirce et al., 2022; *OSWeb*, Mathôt & March, 2022; *lab.js*, Henninger et al., 2022; *Labvanced*, Finger et al., 2017; *Gorilla*, Anwyl-Irvine et al., 2020; *Qualtrics*, Barnhoorn et al., 2015; some server-side solutions: *JATOS*, Lange et al., 2015; *Pavlovia*, Peirce et al., 2022) provide more or less intuitive graphical user interfaces (GUIs) whereby users (researchers) can add, one by one, the building pieces of the experiment (e.g., a text content to appear on the screen or a response required from the participant). The most obvious advantage is that researchers can create ExpApps without any programming knowledge and without having to deal with all the intricacies of the technical implementation. Another advantage is that, typically, such frameworks have dedicated teams (as well as, in case of open-source software, the community) continually working to keep the software top-notch and up to date. In contrast, building web apps without frameworks has so far necessarily been an isolated effort by a single (or a few) individual(s). Part of our goal is to ameliorate this situation by providing guidelines and some template scripts (see below) for such endeavors.

Importantly, certain levels of HTML/CSS/JS knowledge permit the incorporation or management of features and functionalities that are typically not easily achievable or not possible at all through the exclusive use of these GUIs. These include easy control over the entire layout; any custom and complex interaction; intricate conditional operations; tailored restriction of stimulus presentation order; participant access limitations; customized data output; version control; unlimited integration with any third-party technologies and/or JS snippets or libraries; see details in the Appendix. Certain degrees of exceptions to these limitations do exist. Currently, the GUI of Labvanced (Finger et al., 2017) in particular offers remarkably flexible approaches to stimulus ordering and dynamic conditional operations.

There are various degrees of transitions between experiment creator frameworks with GUIs alone and programming web apps without any dedicated frameworks at all. For instance, even when created with GUIs, most frameworks provide the web apps as HTML/CSS/JS files that are modifiable by researchers, though with various degrees of ease. The ease depends on the structure of the scripts, such as how many modifications are required for a given desired change in the experiment, and whether the change(s) in the script will have negative side-effects (e.g., glitches, errors, or other unexpected behavior), and so forth. Some frameworks, for example *OSWeb* and *lab.js*, provide some helpful documentation for such custom modifications. At the extreme, some frameworks, most notably *jsPsych* (De Leeuw, 2015), are explicitly intended for researchers with programming knowledge who merely use the framework as “plugins” (JS libraries), that is, collections of certain utility functions and perhaps some basic script templates that are to be rewritten and/or extended with further code for the specific experiments by the researcher.

The present tutorial is aimed chiefly at those who wish to write their own custom programming scripts for web apps or to adjust or extend, with their own code, templates created by experiment creator frameworks or web apps available elsewhere (e.g., open-source projects or material from other researchers’ experiments). The tutorial and the connected template scripts are not meant to be competing alternatives to experiment creator frameworks – quite the opposite, developers of experiment creator frameworks also benefit from being informed of the latest recommended practices, and may freely follow or borrow from the template. Altogether, we hope to serve all programmers who wish to know how to build, extend, or improve ExpApps.

## Material

This tutorial has three main components. First, a high-level description of the main concepts are given in the present paper, serving as an initial general introduction in particular to those new to creating ExpApps. Second, a specific template for the HTML/CSS/JS implementation is given at https://github.com/gasparl/expapp, which includes typical survey measures (multiple choice questions, scales, etc.) as well as a stop-signal task (SST) as a common example for a psychological response time test. Third, the same repository contains an open-source tutorial, as a Markdown file, which is a greatly extended version of the present paper and includes a variety of detailed technical information and recommendations (specific JS functions, code snippets, etc.). This latter serves as an advanced guide for those in the process of implementing an ExpApp, or those wishing to improve an ExpApp (or a creator framework).

The present paper contains the overarching concepts that are expected to remain unchanged in the foreseeable future. In contrast, the template and the open-source tutorial are intended to be continually updated (by the present authors as well as by the community) based on the development of Internet browsers as well as new software solutions.

Code sections relevant to given topics (e.g., layout, or precise display timing) are marked in the scripts with bracketed numbers as [n1], [n2], and so forth, and are cited via these numbers in the Markdown tutorial (including hyperlinks). All modern text editors for programming allow searching an entire directory with all script files included (as downloaded from the GitHub repository). Hence one has to simply search for, for example, “n2” or “[n2]” (without quotes) to find the relevant functions or lines of code in the given script files, and/or the corresponding description in the open-source tutorial. The GitHub repository can be searched directly too (e.g., as https://github.com/gasparl/expapp/search?q=n2).

Due to its technical nature and despite not going into great detail (and not dwelling on advanced methods such as eye-tracking or 3D simulations), this tutorial necessarily describes a relatively specific approach. Even more so, the template implements one very specific approach. Tasks can be designed and scripts can be written in many different ways, and everyone may have their own preferred implementation. In the end, however, browser-based experimentation is restricted (or enabled) by the functions that the browsers make available. The ways to call these functions so that one makes use of them most effectively is even more restricted. In any case, regardless of personal preferences regarding the details, at the very least the present tutorial demonstrates the general workflow of how one may effectively create web apps for online experiments.

## File structure and web hosting

For an interactive webpage (or: “web app”), there is usually at least one file of each of the following types: (a) HTML (Hypertext Markup Language), the standard language for creating the text content, including very basic formatting, of webpages; (b) CSS (Cascading Style Sheets): a style sheet language for more comprehensive formatting of HTML, and (c) JS (JavaScript): a high-level (“easy”) programming language for making HTML webpages interactive (i.e., allows modifying HTML elements, usually via user interaction). Simply put, one writes a text in HTML, gives it a design with CSS, and then brings it “alive” with JS. Building web apps using such scripts is very intuitive, and there are various excellent free tutorial websites (e.g., W3Schools^2^ or the Mozilla Developer Network^3^) and online courses (e.g., edX^4^) for learning all the basics. For authoritative and up-to-date information on any specific JS method, a most recommended source is the official website of the Mozilla Developer Network (maintained jointly by Mozilla, Google, Microsoft, and Samsung, among others). The rest of this tutorial assumes a basic understanding of these three languages.

Often, there is a single “*index.html*” HTML file. A convenience of this conventional naming is that by entering into a web browser the web address (uniform resource locator; URL) of the directory of the files, without the filename, the web server^5^ by default usually returns the “*index.html*” file, which is subsequently loaded into the browser. Hence, for example, instead of writing https://gasparl.github.io/expapp/index.html, one can just write https://gasparl.github.io/expapp. Often, there is just one CSS file, often named “*style.css*” or similar, that contains all styles for the web page. However, in the template, there is an additional “*rt_task.css*” file, related to the styling of the behavioral part of the experiment, which is somewhat distinct from the rest of the webpage, and therefore may be more conveniently stored and managed in a separate file. Finally, since the template includes a lot of JS code for a variety of different purposes, there are several files to contain these scripts. The most important ones are the following: (a) “*main.js*” – the main workflow of the experiment; (b) “*utils.js*” (within the “*utils*” folder) – various general utility functions; and (c) “*rt_task.js*” (within the “*rt_task*” folder) – JS code related to the behavioral (response time) experiment.

Using such files, one can create a working ExpApp that can be opened and run locally (i.e., on one’s computer) in any modern browser (by opening the “*index.html*” file). However, to conduct an online experiment, there are two crucial further requirements. First, the app should be accessible at the “client side” by users. Here, users mean participants accessing the app via the “World Wide Web” (the Web), that is, online, by entering a given URL in a web browser on any computer with an Internet connection. Second, the collected data should be stored at the “server side” for the researchers. This altogether necessitates a webserver with the capability of storing data via a server-side language such as PHP (PHP: Hypertext Preprocessor; originally: Personal Home Page). For a more detailed theoretical overview of client and server sides, see Grootswagers (2020).

Using web servers and server-side languages may seem daunting at first, but for ExpApps, this is actually very easy. Here, we describe the proper procedure, and, in the template, we provide the necessary code (which can remain almost exactly the same for all experiments; for related details, see the section Data Storage below). Most universities have their own web servers and provide web space for employees and often even for students, either automatically allocated or upon request. ExpApps typically require very little disk storage space, a tiny fraction of typically provided free web spaces, less than a megabyte (though exceptions do exist, e.g., in case of video stimuli; see also, for an informative anecdote, Woods et al., 2015, p. 12). Such small and relatively infrequently accessed^6^ web spaces are also available as free plans of various commercial hosting services (which may be easily found googling “free php web hosting” or similar).

Whichever server may be used, they should have a detailed guide on how to upload files to the server (normally via a file transfer protocol [FTP] client, such as FileZilla, WinSCP, or Krusader). This procedure may differ slightly from server to server, but it is generally no more complicated than logging into an account via a straightforward graphical user interface (of an FTP client) and copying the ExpApp files to the desired directory under the web space (similarly to how one copies files from one directory to another on a computer’s usual local storage space). As soon as the proper files are copied to the server, the ExpApp is accessible at a given URL, and the collected data will be stored at the server. The URL is always provided and described by the web hosting facility. For instance, the root URL of the default personal web space at the University of Vienna is “https://homepage.univie.ac.at/firstname.lastname”. If the “index.html” is placed in the root (top) directory, it will be available via this URL. However, for easier management of multiple projects, each ExpApp’s files may be placed in a separate subdirectory (i.e., folder). For instance, the files for the SST ExpApp could be placed in a subdirectory named “sst_exp.” In that case, the ExpApp (via the “index.html”) will be accessible at “https://homepage.univie.ac.at/firstname.lastname/sst_exp”. As a precaution (for permanent availability, compatibility, etc.), all resources such as third-party plugins and JS libraries (or any media, etc.) should ideally be downloaded by the researcher and also placed under this directory (to be sourced via the “index.html” file).

## Basic layout (HTML/CSS) structure

Having just one HTML file essentially gives a single-page application (SPA). This means that the entire ExpApp will remain, throughout the experimental procedure, at the same URL (e.g., https://homepage.univie.ac.at/gaspar.lukacs/sst_exp). This (a) prevents users arbitrarily navigating back and forth between pages (or even skipping ones, by manually changing the URL); (b) requires only a single-time loading (hence subsequent pages will not require any loading time or even any Internet connection at all); (c) provides an arguably easier-to-maintain, compact code structure; and (d) makes the script straightforward to integrate with JS-based frameworks for creating native (and/or cross-platform) apps. More detailed information about this latter possibility is beyond the scope of the present tutorial. However, there are a variety of free online tutorials for the purpose,^7^ and, given that such frameworks are based on the same HTML/CSS/JS structure as described here, the present tutorial is just as applicable to them regarding ExpApps.

To explore and adjust the style (size, color, etc.) of any HTML element, one can at any time conveniently inspect and manually modify the CSS properties in any major browser’s developer tools. Clicking on any element and selecting the “Inspect” option from the drop-down menu directly displays in the developer tools window’s dedicated tab (e.g., “Inspector” in Firefox or “Elements” in Chrome) the given element’s CSS properties, which can be manually overwritten. Once the desired looks is achieved via the properties modified in the browser (which apply only to the loaded page, and are discarded as soon as the browser tab or page is closed), the same CSS properties can be adjusted accordingly in the local files too.

### Size, color, and font

#### Size

For relatively consistent visual angle (i.e., perceived size) across devices, HTML element sizes should be defined in CSS pixels (px).^8^ This is, importantly, not equivalent to physical display device pixels (whose size varies unpredictably depending on the hardware), but is an abstract unit that explicitly serves to provide a standardized comparable viewing experience across different devices, taking into account typical viewing distances (e.g., smartphones are typically viewed at a closer distance than desktop computers).^9^ For desktop computers (that assume reading at an arm’s length), one CSS pixel should be about 0.26 mm (1/96 inch), but it should be smaller for a laptop computer (ca. 0.20 mm) and smallest for smartphones (ca. 0.16 mm). There may be slight discrepancies between different devices, but uniform appearance and behavior are essential for web pages, and therefore manufacturers can be expected to closely adhere to web standard specifications and conventions.

#### Color

Setting up a desired balance of hue, saturation, and brightness is difficult enough when calibrating the display of colors in a dedicated laboratory (Wilms & Oberfeld, 2018). In online research, however, the large variety of physical devices used to display images together with unpredictable idiosyncratic monitor settings and lighting conditions makes it practically impossible to very accurately render colors, let alone to have identical colors perceived by different participants. Nonetheless, it is reasonable to expect broad color definitions, such as “green” or “red,” and large brightness differences within a screen (i.e., relatively much darker vs. relatively much lighter colors), to hold true for all participants. In other words, it is highly unlikely that, for instance, an element colored fully “green” (in CSS: “#00ff00”) will not be seen by all participants (with full color vision) as some sort of green color, even though the specific type of greenness will differ.

#### Font

If strictly identical font across tests is crucial (or for some reason an unusual font type is needed), one may add font files,^10^ that contain the desired font type outlines, to the app to be loaded (via CSS) and used for all displayed texts. However, in most cases this is probably unnecessary. Practically all operating systems support either Arial (or the near-identical Helvetica) or another sans-serif font type (e.g., on Android, Roboto) that is for the human eye generally hardly distinguishable from Arial. Hence, one may simply specify font family for the entire ExpApp as Arial and a “sans serif” fallback option, so that if Arial is unavailable, a very similar sans serif font type is used.

## Basic operational (JS) structure

Similarly to CSS, any major browser’ developer tools (Console) may be used to very conveniently inspect or modify JS variables too, at any point of the experiment.

### On document load

As soon as the web page (the ExpApp) is loaded in the browser, certain procedures (see below) may be executed as precautions or as preparations of the upcoming experiment.

#### Browser compatibility

“ECMAScript” is a JS standard intended to ensure the interoperability of web apps across different browsers. In 2015, ECMAScript 6 (ES6) was introduced as a major revision, and was soon adapted by all major modern browsers. Nonetheless, web developers often continued to try using JS without ES6 features in order to stay compatible with older browsers. However, given the rapid development of browsers, it should be assumed that nearly all, if not all, users have a browser supporting ES6. Our template therefore includes various ES6 features (however, to be safe, no more recent ones).

#### Query string

Often, there are different versions of the same experiment that differ only in some small but important aspect. For example, the same experiment may be done in different languages. In this case, the language may be indicated via a “query string”: a text attached to the URL, separated by a question mark. For example, to indicate that the given ExpApp should be in English, one may write https://gasparl.github.io/expapp?lg=en, while, for a German version, one may write https://gasparl.github.io/expapp?lg=de. The information for the “lg” parameter can then be accessed in JS. There can be multiple parameters. For instance, https://gasparl.github.io/expapp?lg=de&device=mobile may indicate an ExpApp in German and intended for mobile devices.

#### Multilingual experiments

In case of multiple language versions of the same experiment (e.g., for international and cross-cultural studies), an elegant way to store the text content is to have different JS files for each language (in the template, two such files serve as examples, “*lg_en.js*” for English, “*lg_de.js*” for German), where each contains a JS “dictionary” object with all the various texts of the given ExpApp. On page load, the desired language is detected from the query string (or otherwise defaults to English), the corresponding language file (with the given language’s dictionary) is loaded, and, based on a match between element IDs and the dictionary keys, all texts of the given language are inserted into the HTML.

## Precautionary measures

There are some precautionary measures that may be implemented at any point during the experiment, described in the following sections. One good time for activating these might be right after the participant consented to participation via a button click.

### Unloading the web page

It is easy to leave (technically: “unload”) a web page by closing the browser tab (or window) or by navigating back to a previous page (via the browser’s “Go back one page” or similar button or keyboard shortcut). This could happen, for example, if the participant unwittingly attempts to navigate back to a previous section of the experiment, while this is not in fact permitted (and will not work on a typical single-page app where the URL is unchanged). Therefore, one may disable back navigation and show a warning and ask for confirmation when a participant tries to leave the page.

### Fullscreen

Conducting online experiments throughout using the browser’s fullscreen mode is often recommended and used by experiment creator frameworks, in order to reduce distractions (e.g., Gagné & Franzen, 2023, p. 9), even though it may be debatable whether its impact is substantial. Regardless, the technical implementation is not difficult, although making the browser enter fullscreen mode via JS requires an active user input, such as a keypress or a click on an element. A reasonable approach may be to enter fullscreen when the participant consents on the first page with a button click, and, in case the participant manually exits the fullscreen, to reenter fullscreen whenever the participant continues (via, e.g., a button click or a keypress) to a next section in the experiment. Another option would be, unless fullscreen is a crucial part of the experiment, to respect the participant’s decision not to use fullscreen and allow them to proceed in such a way – likely contributing to a more positive experience.

### Scaling

Preventing page scaling (“zooming” in or out, typically using Ctrl and the mouse wheel) is strongly discouraged by browser vendors and specific programmatic measures have been implemented against it, since they wish all users to indeed be able to scale pages for accessibility (i.e., readability). It should also be considered that, while elaborate methods do exist to measure it (Li et al., 2020), viewing distance is impossible to ensure throughout an online experiments, and therefore different participants may have very different visual angles for the given stimuli anyway. Hence, it may just be better to let them scale the page if they wish to – especially if they otherwise might have difficulty seeing the page content. There is in any case no established method for scaling prevention.

## Data storage

There are several increasingly popular new server-side languages and solutions, but, to date, owing also to the simplicity of its setup, PHP is still by far most popular, alone accounting for the great majority of the market share.^11^ Universities typically provide servers with a PHP interpreter that allows to use PHP code without any necessity for an in-depth understanding of the server’s (or the PHP interpreter’s) workings. Using PHP for ExpApps, the very simple approach that we recommend is to save each participant’s data as a separate text file. The PHP files provided in the template can be used for almost any ExpApp, since essentially all they do is just write any text content data sent from JS to a new file created on the server.

### Partial data

To assess dropout rates (Zhou & Fishbach, 2016), partial data may be intermittently stored. For instance, all data up to the given point may be stored on the server when the page is first loaded, and afterwards on each new section start (new questionnaire, new task, etc.; in case of no clear sections, one may also set up a time interval, e.g., every 5 min). Dropouts are typically discussed in the context of their biasing effects on surveys, but they may in fact impact behavioral data collection as well (Lukács, 2021). Hence, partial data may also be intermittently stored on the server during behavioral data collection at a certain desired interval (e.g., in the template, having about hundred trials altogether, at every tenth trial^12^).

### Complete data

At the end of the experiment, the full complete data may be stored. The JS function awaits the server response and provides corresponding feedback to the participant. In case of successful storage, a success message is shown. In case of any sort of issue (e.g., temporary loss of Internet connection), the participant may be offered options such a retry button via which saving the file at the server is reattempted, and a download button via which the results files may be manually downloaded and sent via email to the researcher.

### Data format

**Fig. 1:**
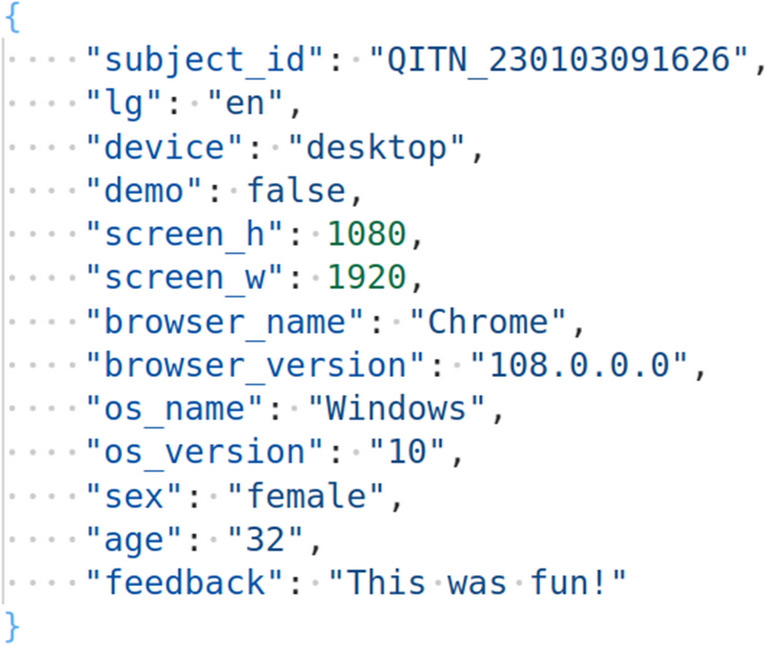
JSON example. *Note*. Sample data from the last, JSON format line from the experiment results of the template ExpApp. The JSON format was “beautified” (reformatted) using Visual Studio Code in order to display each attribute-value pair in a new line, which makes the data even more easy to overview. Such reformatting may also be done in any other code popular editor or via many freely available and easy-to-use online websites for this purpose

Data format may particularly be up to preference, but one convenient way is to keep behavioral data in single lines per trial, while keeping all other, miscellaneous (e.g., demographic) data in JSON (JavaScript Object Notation) format^13^ (Fig. 1) in the very last line of the file. If desired, for subsequent analysis, the JSON format can be easily restructured into newly designated columns per each row.

## Data protection

In the following, we briefly consider data protection primarily from the perspective of EU nations’ ethical conventions (World Medical Association Declaration of Helsinki, 2013) and legal regulations (Regulation [EU], 2016; also known as the General Data Protection Regulation or GDPR). In other parts of the world, the relevant legal frameworks and ethics are generally either comparable to or less stringent than those in the EU (e.g., Greene et al., 2019).

In general, online data collection is subject to the same legal and ethical principles applicable to any other research involving human subjects (Greene et al., 2019; Regulation [EU], 2016): Personal information should be processed lawfully, transparently, and fairly, collected and used only for clearly stated, valid purposes, limited to those relevant purposes, maintained accurately and up-to-date, retained only as long as necessary, and stored securely.

The exact details of certain aspects are debatable (including what constitutes “personal information”), and local laws and institutional regulations must also be taken into account. Nonetheless, in any case, what is relevant from a technical perspective, and hence to the present tutorial, is avoiding the unnecessary or covert collection of personal information, and ensuring the secure storage of any collected data.

Regarding personal information, obvious examples include identity details such as a personal name or birth date, or a participant’s face visibly recorded in a picture or video. It is less clear whether certain types of physiological data (e.g., eye tracking or typing patterns) can be used for personal identification, especially when combined with other types of data. Focusing again on the technical side, browser information (e.g., user agent, plugins), IP (Internet Protocol) addresses, and cookies may each also be categorized as personal information, because, when combined with additional information, they could lead to personal identification (Greene et al., 2019). Therefore, such information should only be collected when truly necessary; when collected, participants should be informed, and the confidential data should be handled securely.

As for the secure handling, our template provides one ideal example of securely saving the data to a (PHP) server. However, it might be necessary to configure the webserver to serve the ExpApp and receive the participant’s data through “Transport Layer Security” (TLS; the modern successor to “Secure Sockets Layer,” SSL), which provides encryption and integrity checks to secure data in transit. For this, the web server needs to be configured with a certificate, which most web space providers can handle for free through the use of Let’s Encrypt (https://letsencrypt.org/). The procedure is specific to each server, but for that very reason the web hosting providers or server administrators normally offer instructions for the implementation, which is usually straightforward. That TLS is in use is indicated in the URL prefix “https://” (where “s” stands for “secure”) in contrast to the plain “http://” URL prefix. Most university web services provide ready-to-use TLS (observable in the “https://” URL prefix by default), so that no configuration is needed by the users.

If alternative approaches are desired or necessary, we recommend consulting an expert. The server space (or database) itself, where the data is stored, should of course also be kept secure (e.g., using a strong password, potentially two-factor authentication, encrypted data, etc.; see, e.g., Jamieson & Salinas, 2018).

## Collecting survey data

Collecting survey data (via checkboxes, multiple choice questions, scales, text input, etc.) is a basic and extremely widely used feature of web apps, and therefore its general setup is mostly common knowledge (e.g., Baatard, 2012), with technical details well described in general HTML tutorials. Precautionary methods for survey data are also extensively discussed in various previous review papers (e.g., Curran, 2016). Nonetheless, in view of ExpApps, one aspect perhaps worth highlighting is that it is helpful to think beyond conventional paper-based checks and to make use of technical possibilities for this purpose. For instance, instead of asking participants to select a specific answer on a Likert scale (which could however also happen by accident), they may rather be asked to click three times on any of the Likert scale items. Some further easy-to-do yet perhaps neglected possibilities in ExpApps are dragging and dropping items, multimedia presentation, and geolocation.

In the context of online experiments, it is advisable (and costs practically nothing), to include a brief question on the calmness and quietness of the environment during testing, and an open-ended question regarding any potential feedback, in particular – and hence the present point’s relevance to this tutorial – if the participant may have experienced any sort of technical issues (especially useful in case of initial piloting).

## Collecting behavioral data

Unlike collecting survey data, collecting behavioral data is not at all a basic feature of web apps, nor is it widely used. Rather, it is largely restricted to those conducting behavioral experiments online, primarily for academic research.

Our template implements keypress and tap response times, but the general approach would similarly apply to other experiments involving sequential stimulus presentation and precise timing. All details of the relevant code, in the “*rt_task.js*” file, are thoroughly commented within the script. Here, only a general outline is given. Namely, a typical and convenient method is to generate, preceding the start of each block, a list of objects where each object contains, as properties, the information for a single trial in the experiment. For example, in the case of a keypress-based Stroop task (where one key is assigned to each color; MacLeod, 1991; Stroop, 1935), it could contain the color name to be displayed, the color in which this name is to be displayed, and the correct key to be pressed. So the object could contain the following property keys: “itemName,” “itemColor,” “correctKey.” Following this, the entire block can be run sequentially taking one object after another from the pre-generated list (until none is left), and, from each object, the desired properties are accessed in order to correctly prepare and execute each given trial. So, in the Stroop task example, a JS function could insert the itemName property in the HTML element to be displayed, and assign its color from the itemColor property. When the keypress response is given by the participant, the pressed key could be checked against the correctKey property to potentially provide feedback and in any case to record whether the response was correct.

Given the lack of supervision in online experiments, it is most recommended to include practice trials with trial-by-trial feedback regarding the correctness of responses (e.g., Gagné & Franzen, 2023). In case of too few valid responses, the entire practice phase may be asked to be repeated. In the template, there is a maximum of one repetition only, and at the second completion the participant is allowed to continue regardless of the ratio of valid responses. This is advisable in case there is a potential concern about habituation, fatigue, or other undesirable effects of overly long practice.

## Undesired participant behavior

The lack of supervision comes with a lack of control too. Therefore, one should ideally record all available relevant information, especially suspicious or undesirable user actions (in which case immediate warnings may also be given to the user). This includes invalid responses (e.g., pressed key not among the ones applicable to the given task), programmatically simulated responses, and unwarrantedly long inactivity at any point during the experiment.

Recent advances in artificial intelligence (AI) deserve special attention. Simple bots can be programmed to automatically fill out surveys. AI algorithms can scrape the web to gather information or specific answers to questions in the experiment. Advanced natural language processing models can provide convincing replies to open-ended questions. There is no perfect assurance against AI use. Nonetheless, mass submission via simple bots (in case of a publicly available survey) can be greatly mitigated by CAPTCHAs (e.g., Dinh & Hoang, 2023), potentially complemented by detecting suspicious browser characteristics (such as the limited features of headless browsers that bots tend to use, e.g., no plugins or APIs). One may also analyze basic expected patterns, such as time of completion and consistency among the individual’s responses. For open-ended questions’ reply fields, paste and drop actions can be disabled to hinder the use of AI-generated text – although adept programmers can circumvent this.

There is plenty of empirical evidence that many online participants cheat when they have motivation and opportunity (e.g., Nagin & Pogarsky, 2003). However, studies developing and investigating preventive measures have been scarce, and, to our knowledge, all focus on information search (i.e., participants searching the Internet for the correct answers in a survey). To this latter issue, proposals for prevention and detection include setting time limit (Domnich et al., 2015), asking the participant to pledge not to cheat (Clifford & Jerit, 2016), and detecting the survey being obscured by another window or application (Diedenhofen & Musch, 2017) – such approaches may to a great degree curtail information search, but do not fully prevent it (see Graham, 2023).

A systematic empirical investigation of other kinds of cheating seem well overdue. Speaking from abundant personal experience, some participants are really capable of all sorts of trickery to get done with the task faster or easier. To mention one typical phenomenon: in case of multiple-page apps, participants will manually navigate to the last page to get the completion code. But participants’ endeavors can go much further. In one case (using a single-page ExpApp, and completion code stored only at the server), we observed missing data in a supposedly completed file. We contacted the participant, who then admitted having waded through our code to find the submission mechanism, using which he submitted his incomplete data as a supposedly completed file (which triggered the return of the completion code). At least in this and similar cases, the cheater can be detected based on incomplete data. However, again, there is no perfect assurance against cheating. For instance, participants may use an external robotic lever for responses, or they may just generate artificial data to be submitted – though such great efforts to substitute valid participation in a brief task seems unlikely.

Importantly, non-compliance has been observed far less often on Prolific than on more general crowdsourcing websites for microtasks (MTurk, Appen) – also corresponding to the conclusions of formal assessments of data quality per platform (see, e.g., Peer et al., 2021; Uittenhove et al., 2022).

## Pretesting

Conventional software development normally involves programmatic automatized unit-testing that checks whether certain parts of the given software keep their intended behavior following each modification of the code. Web-based interfaces are however not straightforward to unit test in the first place (though, for a popular software solution, see, e.g., Jasmine with Karma). Even so, in case of ExpApp creator frameworks, it might be useful to implement fully automatized testing that can in a uniform manner verify each newly created ExpApp to detect basic errors. De Leeuw et al. (2023) provide a comprehensive overview and practical demonstration of this approach as well as of the related technical possibilities.

However, regarding custom-made ExpApps, in case of modifications from one experiment to another, the intended outcomes may differ to such an extent that it is more difficult to follow up on correcting the unit tests (let alone writing them in the first place; see De Leeuw et al. 2023, p. 1864) rather than to simply manually test it all again – which in any case seems necessary as unit tests cannot be fully relied upon. Therefore, in most cases, it probably makes more sense to just thoroughly manually pretest the app for each new experiment via several browsers. For semi-manual testing however, user actions such as keypresses can be simulated via dedicated JS functions. This is particularly relevant to behavioral data collection, where a lengthy and strenuous examination, such as hundreds of trials measuring key responses, can be run with automatized simulations of keypresses.

For any ExpApp, a simplified, demo version may be created, which can be more easily run than the full version. For instance, in our template, the changes compared to the full version are: (a) there are less trials per block (just one per each unique type); (b) none of the questions are obligatory in order to proceed to the next page (and there is no alert if questions are not answered); (c) no fullscreen is initiated automatically; (d) no data are saved on the server. This is, on the one hand, to serve as a demonstration for collaborators, reviewers, and the eventual readers of a published study. On the other hand, it may also serve to more easily pretest the main features of an ExpApp. As explained above, the demo version of the ExpApp can be indicated in the URL’s query string that is subsequently detected in JS.

**Fig. 2:**
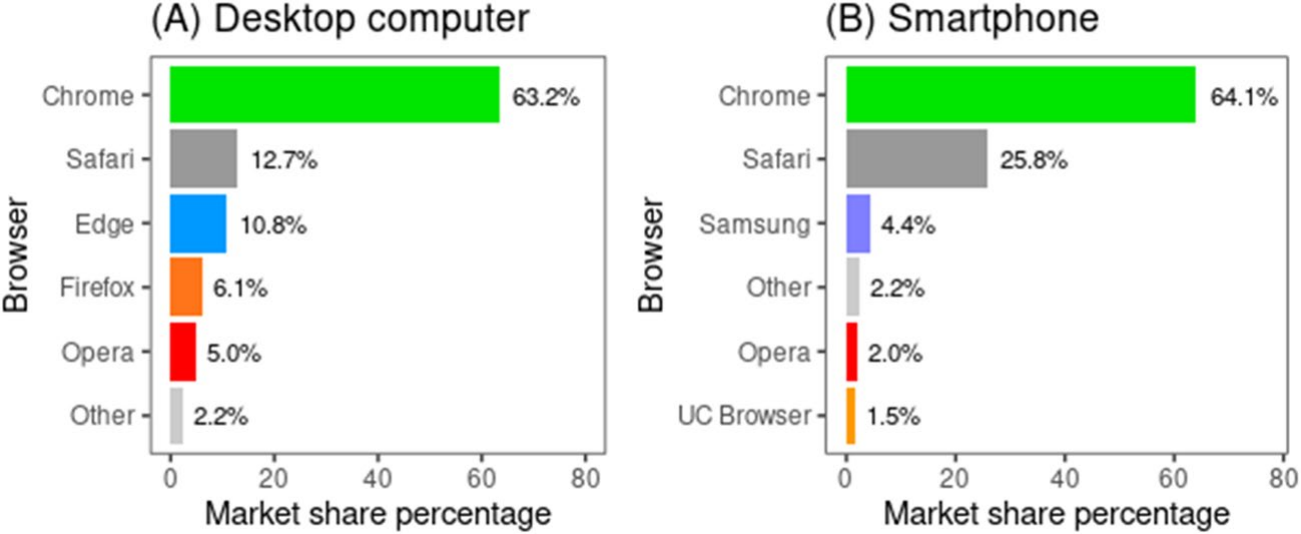
Browser market share for desktop computers and for smartphones. *Note*. Estimated browser market share in October 2023. (Data from https://gs.statcounter.com/browser-market-share/; accessed on November 21, 2023)

Again, uniform behavior (based on the specifications by the World Wide Web Consortium) is essential for web apps, and very unlikely to be ignored by vendors of software and hardware. Even so, it has been repeatedly shown that different combinations of operating systems and browsers may give slightly different results (e.g., Bridges et al., 2020), and browsers in particular differ in their implementation and support of JS methods. To prioritize pretesting, one may first off rely on market share statistics that also indicate the likely distribution of the types of the given software and hardware of eventual participants (Anwyl-Irvine et al., 2021). For one, the current browser market share is almost entirely (> 96%) covered by Google Chrome, Safari, Microsoft Edge, Mozilla Firefox, Samsung Internet, and Opera, with the first (Google Chrome) covering the majority (> 60%), and first four covering the vast majority (> 90%).^14^ However, it is also good to keep in mind that with the exception of Safari and Mozilla Firefox, all these browsers are based in a large part on the (itself alone infrequently used) Chromium browser. Furthermore, Chromium itself is partly based on Safari’s (WebKit) code. The implication is that, at least for desktop computers and laptops (see Fig. 2), the first browser to pretest is Google Chrome, since not only does this cover the majority, but it is also a likely indication of how other Chromium-based browsers (and, to a lesser degree, Safari) work. The next would be Mozilla Firefox, due to its standalone codebase; then, Safari, due to its relatively large coverage and relatively independent codebase. Doing pretests on the rest of the major browsers as well may still be useful.

Finally, however, if uniform appearance and behavior is crucial, one may disallow participation using any browser except the desired one(s). Participants may be warned and prevented from continuing in case of the detection of any undesired browser.

## Concluding notes

The present tutorial gave an overview of the main aspects of creating online psychological experiments via the HTML/CSS/JS framework. We highlighted key considerations in respect of the latest technical developments and challenges, including some neglected aspects of online research. Although our recommendations and implementations present one rather specific approach to creating ExpApps, they altogether also serve as a practical roadmap for leveraging browser capabilities in online experiments efficiently. Regardless of one’s preferred methods, this tutorial provides a foundational understanding of the technical workflow necessary to develop versatile, robust, and customizable ExpApps. By equipping researchers with these technical insights, we aim to enhance the quality and expand the scope of online psychological experiments, also contributing to the broader scientific discourse.

This tutorial is by no means a definitive encyclopedia for all technical details. However, integrally connected to the present paper, a specific implementation is also given via the “living” open-source template project at https://github.com/gasparl/expapp, including an open-source tutorial in a Markdown file that contains more extensive and advanced technical details. The contents of this repository are all completely free to copy, modify, and use for any future experiment or any other purpose. Both the scripts and the tutorial in the repository are intended to be continually improved by the community so that they will always follow the latest technological advancements and empirical findings.

## Data Availability

All material is available via https://github.com/gasparl/expapp (10.5281/zenodo.7750017).

## Appendix

### Advantages of custom coding for ExpApps

As briefly explained in the introduction, some GUI-based ExpApp creators have remarkable capabilities. Nonetheless, in general, knowledge of the HTML/CSS/JS framework can permit or facilitate a variety of important features and functionalities.

With CSS alone, one has easy control over the entire layout. It is straightforward to dynamically set or change the size of any element and/or the color of any single pixel in any moment of the experiment via CSS, as well as to test and explore possibilities in real-time throughout the various phases of the experiment (via browser developer tools, explained later in the tutorial). GUIs would typically require separately creating dedicated objects or images for each layout change in each phase or trial of an experiment. Considering, for example, a complicated new experiment (with many complex stimuli) that still requires exploration and many changes while being created, this can be an extremely laborious, repetitive, taxing procedure. Creating a great number of similar but in certain ways different stimuli (e.g., positioned in different parts of the screen) is particularly time-consuming with GUIs, while it is minimal effort in programming (by, e.g., adding incremental modifications of the same element base in a loop).

Custom JS makes possible any custom and complex interaction, such as, for example, dragging elements or clicking on moving and changing elements. GUIs normally only allow basic input such as keypresses or clicks on fixed objects.

JS also allows complex processing for conditional operations. For example, a specific *A* versus *B* follow-up task might be chosen depending on slower versus faster response times in an initial task. Another important example is when participants select their own stimuli in an initial task (e.g., choosing, out of 20 photos, the five most attractive ones), which are then to be presented to them in a follow-up task. Relatedly, one can restrict presentation order in any desired way. While frameworks typically offer some overall randomization procedure for the order of presenting a group of stimuli, they do not offer more specific rules such as, for example, “item *X* must never follow item *Y*” or “following every second presentation of item *X*, item *Z* should be presented next.”

One may introduce custom restriction of participation via JS (potentially complemented by some server-side code for more security). For example, one may allow participation only in a specific time frame (e.g., 10–12 AM in the participant's time zone, or any given calendar days). This can be a vital, for example, for longitudinal studies, where the follow-up experiment can be programmatically restricted to the required time frame given participants’ times of completion of the preceding experiment.

Via JS, researchers may format the experiment’s outcome data exactly as they want, and as it is most convenient for their subsequent analysis. This may also include some preprocessing and/or statistical calculations, especially when this is needed for providing feedback to the participant at the end of the examination (e.g., evaluation scores, etc.).

Using code independent of GUIs straightforwardly allows version control (e.g., via *git*, using GitHub or GitLab), with which one can easily track changes in the web app and recombine parts of different versions (e.g., when just one specific part of the app turns out to be problematic/undesirable and should be reverted to an earlier version).

Finally, custom code allows unlimited integration with any third-party technologies and/or JS snippets or libraries (e.g., game engines, psychophysiology tools), including frameworks for creating native applications for smartphone or desktop operating systems (which opens up possibilities of native behavior, e.g., easy access to and control of hardware, push notifications, etc.; see, e.g., Singh & G, 2021). Currently, experiment creators seem to lag behind in supporting portable devices and touchscreens. With a little knowledge of the HTML/CSS/JS framework, ExpApps can be easily customized to accommodate smartphones and tablets.

## Footnotes

1 A software *stack* is a collection of technology components that work together to support the execution of a particular application (here: a web app).

2 https://www.w3schools.com/

3 https://developer.mozilla.org/en-US/docs/Learn/Getting_started_with_the_web

4
https://www.edx.org/

5 A web server consists of the hardware where the web content (here: the ExpApp files) is stored and the software that makes the web content accessible to Internet users (usually via web browsers). For more, see, for example, https://developer.mozilla.org/en-US/docs/Learn/Common_questions/Web_mechanics/What_is_a_web_server.

6 Experiments with substantial material to download and/or with many thousands of participants may exceed free tier traffic limits, but this is not typical for online psychological experiments.

7 Mainly via the official websites of the relevant framework (e.g., Native React or Ionic).

8 The notable exception is when an element’s size is specified relative to its parent element’s size. For instance, a smaller square within a larger square may be specified to have height and width as 50%, so that it would always be half the size of the larger square (whose width and height is specified in pixels). In that case, both can be modified by modifying the latter’s pixel sizes

9 For a detailed explanation, see https://hacks.mozilla.org/2013/09/css-length-explained/.

10 There are countless free font files (WOFF, TTF, etc.), for various font types, available online, and easy to integrate with CSS.

11 https://w3techs.com/technologies/history_overview/programming_language/ms/y

12 It could also be done on every single trial, but that seems unnecessary in terms of dropout estimates, and would increase server load by ten times. While uploading small text files is a very light job for a regular server, caution is advisable, in particular when considering the potentially simultaneous participation of hundreds of participants.

13 JSON is a standard data format that uses human-readable text to store and transmit data objects consisting of attribute-value pairs and arrays. Originally based on JavaScript’s object syntax, today it is also in widespread use in all kinds of applications unrelated to JavaScript.

14 Source: https://gs.statcounter.com/browser-market-share. However, for real experiments, location and device type should also be taken into account (see, e.g., https://www.similarweb.com/browsers/worldwide/mobile-phone/).
